# Impact of the Implementation of the Affordability Care Act on Gastric Cancer Survival Rates

**DOI:** 10.7759/cureus.64139

**Published:** 2024-07-09

**Authors:** Oluwasegun A Akinyemi, Oluwatayo Awolumate, Mojisola E Fasokun, Eunice Odusanya, Oluwatobi Lasisi, Derek Ugwendum, Terhas Asfiha Weldeslase, Oluranti O Babalola, Funmilola M Belie, Miriam Micheal

**Affiliations:** 1 Health Policy and Management, University of Maryland School of Public Health, College Park, USA; 2 Surgery, Howard University College of Medicine, Washington, D.C., USA; 3 Internal Medicine, Howard University College of Medicine, Washington, D.C., USA; 4 Epidemiology and Public Health, University of Alabama at Birmingham, Birmingham, USA; 5 Obstetrics and Gynecology, Howard University College of Medicine, Washington, D.C., USA; 6 Family Medicine, Howard University College of Medicine, Washington, D.C., USA; 7 Internal Medicine, Richmond University Medical Center, Staten Island, USA; 8 Social Work, University of South Carolina, Columbia, USA; 9 Public Health, Southern Connecticut State University, New Haven, USA; 10 Internal Medicine, University of Maryland School of Medicine, Baltimore, USA

**Keywords:** causal inference, difference-in-difference analysis, medicaid expansion, cancer-specific survival (css), affordable care act, gastric cancer

## Abstract

Introduction

Gastric cancer, a significant public health concern, remains one of the most challenging malignancies to treat effectively. In the United States, survival rates for gastric cancer have historically been low, partly due to late-stage diagnosis and disparities in access to care. The Affordable Care Act (ACA) sought to address such disparities by expanding healthcare coverage and improving access to preventive and early treatment services.

Objective

This study aims to determine the causal effects of the ACA's implementation on gastric cancer survival rates, focusing on a comparative analysis between two distinct U.S. states: New Jersey, which fully embraced ACA provisions, and Georgia, which has not adopted the policy, as of 2023.

Methods

In this retrospective analysis, we utilized data from the Surveillance, Epidemiology, and End Results Program (SEER) registry to assess the impact of the ACA on cancer-specific survival (CSS) among gastric cancer patients. The study spanned the period from 2000 to 2020, divided into pre-ACA (2000-2013) and post-ACA (2016-2020) periods, with a two-year washout (2013-2015). We compared Georgia (a non-expansion state) to New Jersey (an expansion state since 2014) using a Difference-in-Differences (DiD) approach. We adjusted for patient demographics, income, metropolitan status, disease stage, and treatment modalities.

Results

Among 25,061 patients, 58.7% were in New Jersey (14,711), while 41.3% were in Georgia (10,350). The pre-ACA period included 18,878 patients (40.0% in Georgia and 60.0% in New Jersey), and 6,183 patients were in the post-ACA period (45.2% in Georgia and 54.8% in New Jersey). The post-ACA period was associated with a 20% reduction in mortality hazard among gastric cancer patients, irrespective of the state of residence (HR = 0.80, 95% CI: 0.73-0.88). Patients who were residents of New Jersey experienced a 12% reduction in mortality hazard compared to those who resided in Georgia in the post-ACA period (HR = 0.88, 95% CI: 0.78-0.99). Other factors linked to improved survival outcomes included surgery (OR = 0.30, 95% CI: 0.28-0.34) and female gender (OR=0.83, 95% CI: 0.76-0.91).

Conclusion

The study underscores the ACA's potential positive impact on CSS among gastric cancer patients, emphasizing the importance of healthcare policy interventions in improving patient outcomes.

## Introduction

Gastric cancer, also known as stomach cancer, poses a significant challenge in oncology, accounting for about 1.5% of all new cancer cases annually in the United States [1]. The American Cancer Society estimates that in 2024, there will be approximately 26,890 new cases (16,160 in men and 10,730 in women) and 10,880 deaths (6,490 men and 4,390 women) due to gastric cancer [1]. Characterized by a high mortality rate and often diagnosed at a late stage, survival rates for gastric cancer are distressingly low, with a five-year relative survival rate of 33% to 36% for all stages of gastric cancer combined [2,3]. In addition, the economic impact is also substantial, with the cost of care for gastric cancer in the United States estimated at $2.31 billion in 2024 [4]. These figures underscore the urgent need for improved detection and treatment strategies.

Significant disparities in gastric cancer incidence, treatment, and survival are evident, particularly among racial and ethnic minorities and individuals from lower socioeconomic backgrounds [4-6]. These disparities highlight a critical gap in our healthcare system's ability to provide equitable cancer care.

The Affordable Care Act (ACA), enacted in 2010, represents a landmark effort to expand healthcare access, improve health outcomes, and reduce healthcare disparities across the United States. [7,8]. While the ACA has broadly impacted healthcare, leading to earlier cancer diagnoses, increased access to treatment, and improved survival rates among cancer patients [9-11], its effects on specific cancer types, especially gastric cancer, have not been thoroughly investigated.

These disparities highlight a critical gap in our healthcare system's ability to provide equitable cancer care. Despite the potential of the ACA, particularly its Medicaid expansion component [12], to address these disparities by enhancing healthcare access for underserved populations [13-15], the specific impact of the ACA on gastric cancer survival rates remains underexplored. This study gap is significant because understanding the ACA's effects on gastric cancer can inform future policy interventions aimed at reducing healthcare disparities and improving outcomes for a cancer type that disproportionately affects vulnerable populations.

This study aims to assess the causal effects of the ACA's implementation on gastric cancer survival rates through a Difference-in-Difference (DiD) analysis of two U.S. states: New Jersey, which fully embraced ACA provisions, and Georgia, which has not adopted the policy as of 2023. By comparing gastric cancer survival rates before and after the ACA's implementation between these states, this research seeks to illuminate the potential role of the ACA in mitigating disparities in gastric cancer care and outcomes. This approach will fill a significant research gap and contribute to a more nuanced understanding of how healthcare policy can influence cancer survival rates in the United States.

## Materials and methods

Study design

This study employs a retrospective cohort design using Surveillance, Epidemiology, and End Results (SEER) registry data [16] to evaluate the impact of the ACA on cancer-specific survival (CSS) among gastric cancer patients. SEER is an authoritative source of information on cancer incidence and survival in the United States and includes demographic information, primary tumor site, tumor morphology, stage at diagnosis, first course of treatment, and follow-up for vital status, covering approximately 34.6% of the U.S. population. The analysis spans from 2000 to 2020, segmented into pre-ACA (2000-2013) and post-ACA (2014-2020) periods.

Study population

The study population consists of patients diagnosed with gastric cancer. We used the third edition of the International Classification of Diseases for Oncology (ICD-O-3), which categorizes gastric cancer as C16.0-C16.9, a classification validated by the SEER database. Patients were included if diagnosed within the study periods and were either Georgia or New Jersey residents. Georgia represents a non-expansion state, while New Jersey is an expansion state having implemented Medicaid expansion under the ACA in 2014. The selection of these states facilitated a comparative analysis of the impact of Medicaid expansion on gastric cancer outcomes.

Data collection

Data were extracted from the SEER 17 Registries Database. The SEER*Stat Database, released in April 2023, contains cancer incidence data from 17 registries covering 2000-2020, linked to county attributes from 1990 to 2021. Managed by the National Cancer Institute, this database provides insights into cancer trends and disparities across U.S. counties. Variables of interest included patient demographics (age, race, marital status), socioeconomic status indicators (household median income), clinical characteristics (disease stage at presentation), treatment modalities (surgery, chemotherapy), and outcomes, including disease stage at presentation and CSS.

Variable of interest

The implementation of the ACA was operationalized as the categorical variable representing two time periods, pre-ACA (2000-2013) and post-ACA (2016-2020), with a two-year washout period (2013-2015) to account for the transitional phase post-ACA implementation. Additionally, the interaction between the ACA implementation and the expansion status (Georgia vs. New Jersey) was examined to assess the differential impact of Medicaid expansion on gastric cancer outcomes across states.

Primary outcomes of interest

These include the disease stage at presentation and CSS.

Disease Stage at Presentation

One of the primary outcomes of interest was the disease stage at the time of gastric cancer diagnosis. The disease stage was categorized into three groups: localized (confined to the stomach), regional (spread to nearby lymph nodes or tissues), and distant metastasis (spread to distant organs or tissues).

CSS

The CSS was a crucial indicator of long-term prognosis and treatment effectiveness among cancer patients. The CSS was calculated using a Cox regression analysis. The Cox regression, also known as the proportional hazards model, is a statistical technique used to examine the association between the time until an event occurs (cancer-specific mortality) and various predictor variables (states stratified by expansion status (Georgia vs. New Jersey) and period (pre-ACA vs. post-ACA).

Covariates

Demographics, including age at diagnosis, gender, marital status, and race/ethnicity (classified as non-Hispanic White, non-Hispanic Black, Hispanic, and Others), were covariates. Socioeconomic status was assessed using the patient household median income, categorized as <$70,000 and ≥$70,000, and the metropolitan status of the area of residence. Clinical variables such as stage at diagnosis (localized, regional, distant metastasis) and initial treatment modalities (surgery, chemotherapy, radiotherapy) were also considered.

Statistical analysis

Descriptive statistics, including means, medians, and standard deviations, were used to summarize the characteristics of the study population. Specifically, we utilized Chi-square tests to examine the distribution of categorical variables across pre- and post-ACA periods. To quantify differences in gastric cancer outcomes between Georgia and New Jersey, a DiD analysis was conducted. This analysis compared changes in disease stage at presentation and CSS before and after ACA implementation, both within and between the states, while adjusting for covariates such as patients' age, race, income, disease stage, and treatment modalities. Additionally, we used margins analysis to calculate the predicted probabilities, which enhances the interpretation of the interaction term by illustrating the probabilities of each outcome across states and periods, thereby providing a clearer understanding of the impact. Statistical significance was assessed using two-tailed tests with a predetermined alpha level of p < 0.05. All analyses were performed using the STATA 16 statistical software package (StataCorp LLC, College Station, TX, USA).

## Results

Table 1 highlights the baseline characteristics of the study population (N = 25,061), which showed significant differences between pre- and post-ACA implementation across several variables. The pre-ACA period spanned from 2000 to 2013, and the post-ACA period extended from 2014 to 2020. The ACA became fully operational in New Jersey in January 2014. The average age of the population was slightly lower post-ACA (64.9 ± 1.0 years) compared to pre-ACA (65.2 ± 12.5 years). There was a higher percentage of patients from Georgia in the post-ACA period (45.2%) compared to pre-ACA (40.0%), while New Jersey saw a decrease from 60.0% to 54.8% (p < 0.001). The racial composition also changed significantly, with an increase in Hispanic patients (12.8% vs. 9.3%) and a significant drop in non-Hispanic whites post-ACA (61.2% vs. 54.9%). The disease stage at presentation showed a higher percentage of localized cases post-ACA (36.4% vs. 32.0%) and fewer regional cases (26.3% vs. 31.7%, p < 0.001). Income distribution remained similar. Marital status changes included increased single and married individuals post-ACA (p < 0.001). The use of chemotherapy increased post-ACA (48.1% vs. 39.8%, p < 0.001), while surgery and radiation saw slight decreases, respectively.

**Table 1:** Baseline characteristics of the study population Note: Summary statistics are mean ± standard deviation (SD), or n (%) ACA: Affordable Care Act

Variable	Total population (N = 25,061)	Pre-ACA (n = 18,878)	Post-ACA (n = 6,183)	p-value
Age (years)	65.1 ± 12.8	65.3 ± 12.9	64.6 ± 12.5	<0.001
States by policy implementation status				<0.001
Georgia	10,350 (41.3%)	7,556 (40.0%)	2,794 (45.2%)	
New Jersey	14,711 (58.7%)	11,322 (60.0%)	3,389 (54.8%)	
Females	10,057 (40.1%)	7,558 (40.0%)	2,499 (40.0%)	0.596
Race				<0.001
White	14,957 (59.7%)	11,560 (61.2%)	3,397 (54.9%)	
Black	5,964 (23.8%)	4,445 (23.6%)	1,519 (24.6%)	
Hispanic	2,555 (10.2%)	1,762 (9.3%)	793 (12.8%)	
Others	1,585 (6.3%)	1,111 (5.9%)	474 (7.7%)	
Grade				<0.001
Localized	5,775 (33.4%)	3,810 (32.0%)	1,965 (36.4%)	
Regional	5,190 (30.0%)	3,770 (31.7%)	1,420 (26.3%)	
Distant	6,338 (36.6%)	4,326 (36.3%)	4,326 (36.3%)	
Household median income				0.591
< $70,000	12,484 (49.8%)	9,385 (49.7%)	3,099 (50.1%)	
≥ $70,000	12,572 (50.2%)	9,488 (50.3%)	3,084 (49.9%)	
Marital status				<0.001
Divorced	1,898 (8.4%)	1,393 (8.1%)	505 (9.3%)	
Married	13,026 (57.3%)	9,856 (57.0%)	3,170 (58.3%)	
Single	3,473 (15.3%)	2,506 (14.5%)	967 (17.8%)	
Widowed	4,342 (19.1%)	3,544 (20.5%)	798 (14.7%)	
Treatment				
Chemotherapy	10,493 (41.9%)	7,518 (39.8%)	2,975 (48.1%)	<0.001
Surgery	11,775 (54.1%)	9,096 (56.0%)	2,679 (48.3%)	<0.001
Radiation	4,856 (19.6%)	3,744 (20.1%)	1,112 (18.3%)	<0.001

Table 2 is a Cox regression analysis predicting mortality hazard in individuals with gastric cancer in the study period. The interaction between the ACA implementation period and states based on the policy implementation status was substantive. Patients treated in New Jersey in the post-ACA implementation period of 2014-2020 had a reduced mortality hazard (HR = 0.881, 95% CI: 0.778-0.998, p = 0.047) compared to those treated in Georgia. Other significant predictors included staging, surgery, radiotherapy, gender, chemotherapy, and age. The mortality hazard was also significantly lower post-ACA implementation (HR = 0.800, 95% CI: 0.731-0.875, p < 0.001), irrespective of the states.

**Table 2:** Cox regression predicting the mortality hazards for patients with gastric cancer (SEER registry 2000-2020) Reference is the distant metastasis at presentation ACA: Affordable Care Act; CI: Confidence interval; SEER: Surveillance, epidemiology, and end results

Variable	Hazard ratio	Standard error	z	p-value	Lower CI	Upper CI
Age (years)	1.01	0.001	8.5	<0.001	1.008	1.012
Female	0.905	0.026	-3.48	0.001	0.856	0.958
Race/ethnicity						
White	Reference					
Black	0.944	0.031	-1.74	0.081	0.885	1.007
Hispanic	0.979	0.044	-0.47	0.636	0.897	1.069
Asian/Pacific Islanders	0.877	0.049	-2.34	0.019	0.786	0.979
Others	1.638	0.671	1.21	0.228	0.734	3.655
Marital status						
Divorced	Reference					
Married	0.915	0.042	-1.92	0.055	0.836	1.002
Single	1.099	0.059	1.76	0.079	0.989	1.221
Widowed	0.999	0.057	-0.01	0.992	0.893	1.119
Household median income						
< $70,000	Reference					
≥ $70,000	1.026	0.03	0.9	0.369	0.97	1.086
Disease stage at presentation						
Regional	5.126	0.237	35.33	<0.001	4.682	5.613
Distant	8.395	0.407	43.88	<0.001	7.634	9.232
Treatment						
Surgery	0.371	0.012	-29.48	<0.001	0.348	0.397
Radiotherapy	1.083	0.035	2.48	0.013	1.017	1.154
Chemotherapy	0.664	0.021	-12.87	<0.001	0.624	0.707
ACA implementation period						
Pre-ACA implementation	Reference					
Post-ACA implementation	0.8	0.037	-4.87	<0.001	0.731	0.875
States by policy implementation status						
Georgia	Reference					
New Jersey	0.86	0.028	-4.69	<0.001	0.808	0.916
Interaction between states and policy implementation status						
Georgia X post-ACA period	Reference					
New Jersey X post-ACA period	0.881	0.056	-1.99	0.047	0.778	0.998

**Figure 1:**
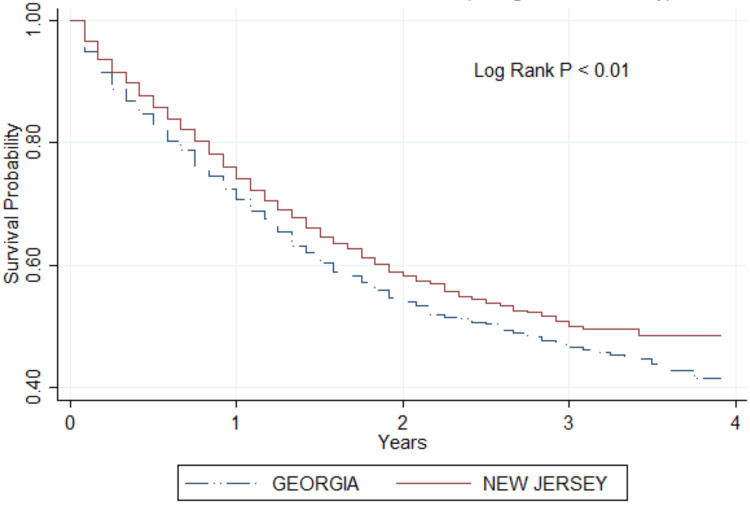
Kaplan-Meier curve showing gastric cancer survival between Georgia and New Jersey in the post-ACA period p < 0.01 shows that the difference in survival between the two states in the post-ACA period is statistically significant ACA: Affordable Care Act

The Kaplan-Meier survival curve in Figure 1 illustrates the survival probabilities for gastric cancer patients in Georgia versus New Jersey in the post-ACA period. The graph indicates a consistent difference in survival between the two states, with New Jersey showing higher survival probabilities in the post-ACA period. At the end of a four-year follow-up period, the survival probability in New Jersey is notably higher than in Georgia. The Log Rank test, with a p-value less than 0.01, confirms that this difference is statistically significant. 

The multinomial logistic regression analysis (Table 3) showed that individuals were more likely to present with localized gastric cancer in the post-ACA implementation period (RR ratio: 1.204, p = 0.025) compared to the pre-ACA period. The relative risk was lower for patients in New Jersey compared to Georgia (RR ratio: 0.847, p = 0.021). Additionally, there was a significant interaction effect between the state and policy implementation period, with individuals in New Jersey post-ACA being more likely to present with localized gastric cancer compared to those in Georgia post-ACA (RR ratio: 1.474, p = 0.001).

**Table 3:** Multinomial logistic regression predicting the relative risk of an individual presenting with localized gastric cancer (SEER registry 2000-2020) Reference is the distant metastasis at presentation. Each relative risk ratio represents the risk of an individual presenting with localized disease versus distant metastasis ACA: Affordable Care Act; CI: Confidence interval; SEER: Surveillance, epidemiology, and end results

Variable	Relative risk ratio	Standard error	z	p-value	Lower CI	Upper CI
Age (years)	1.007	0.002	2.86	0.004	1.002	1.011
Female	1.196	0.069	3.1	0.002	1.068	1.339
Race/ethnicity						
White	Reference					
Black	0.889	0.06	-1.73	0.083	0.778	1.015
Hispanic	0.898	0.082	-1.17	0.241	0.75	1.075
Asian/Pacific Islanders	0.881	0.097	-1.14	0.253	0.71	1.095
Others	0.392	0.368	-1	0.319	0.062	2.469
Marital status						
Divorced	Reference					
Married	0.923	0.087	-0.85	0.396	0.766	1.111
Single	0.863	0.095	-1.34	0.179	0.696	1.07
Widowed	0.873	0.102	-1.16	0.247	0.694	1.098
Household median income						
< $70,000	Reference					
≥ $70,000	0.925	0.057	-1.28	0.2	0.82	1.042
Treatment						
Surgery	18.839	1.102	50.21	<0.001	16.799	21.126
Radiotherapy	1.516	0.12	5.26	<0.001	1.298	1.77
Chemotherapy	0.184	0.011	-27.53	<0.001	0.163	0.207
ACA implementation period						
Pre-ACA implementation	Reference					
Post-ACA implementation	1.204	0.099	2.25	0.025	1.024	1.415
States by policy implementation status						
Georgia	Reference					
New Jersey	0.847	0.061	-2.31	0.021	0.735	0.975
Interaction between states and policy implementation status						
Georgia X post-ACA period	Reference					
New Jersey X post-ACA period	1.474	0.168	3.41	0.001	1.179	1.843

The multinomial logistic regression analysis (Table 4) reveals that the ACA implementation period was associated with a decreased risk of individuals presenting with regional gastric cancer compared to the pre-ACA period (RR ratio: 0.789, p = 0.006). There was no significant difference in the risk between New Jersey and Georgia (RR ratio: 0.980, p = 0.777). However, the interaction between the state and policy implementation period indicates a higher risk of regional gastric cancer presentation in New Jersey post-ACA compared to Georgia post-ACA (RR ratio: 1.284, p = 0.032).

**Table 4:** Multinomial logistic regression predicting the relative risk of an individual presenting with regional gastric cancer (SEER registry 2000-2020) Reference is the distant metastasis at presentation. Each relative risk ratio represents the risk of an individual presenting with localized disease versus distant metastasis ACA: Affordable Care Act; CI: Confidence interval; SEER: Surveillance, epidemiology, and end results

Variable	Relative risk ratio	Standard error	z	p-value	Lower CI	Upper CI
Age (years)	1.02	0.002	8.37	<0.001	1.015	1.025
Female	0.929	0.054	-1.25	0.21	0.828	1.042
Race/ethnicity						
White	Reference					
Black	1.103	0.075	1.44	0.15	0.965	1.262
Hispanic	1.147	0.104	1.52	0.128	0.961	1.369
Asian/Pacific Islanders	1.017	0.112	0.15	0.878	0.819	1.263
Others	0.264	0.309	-1.14	0.255	0.027	2.612
Marital status						
Divorced	Reference					
Married	0.903	0.087	-1.07	0.287	0.748	1.09
Single	1.051	0.117	0.45	0.656	0.845	1.307
Widowed	1.054	0.126	0.44	0.657	0.835	1.332
Household median income						
< $70,000	Reference					
≥ $70,000	1.081	0.065	1.29	0.196	0.96	1.217
Treatment						
Surgery	19.78	1.147	51.49	<0.001	17.656	22.16
Radiotherapy	3.735	0.247	19.97	<0.001	3.282	4.251
Chemotherapy	1.129	0.069	2	0.046	1.002	1.272
ACA implementation period						
Pre-ACA implementation	Reference					
Post-ACA implementation	0.789	0.068	-2.77	0.006	0.667	0.933
States by policy implementation status						
Georgia	Reference					
New Jersey	0.98	0.069	-0.28	0.777	0.853	1.126
Interaction between states and policy implementation status						
Georgia X post-ACA period	Reference					
New Jersey X post-ACA period	1.284	0.149	2.15	0.032	1.022	1.612

Table 5 shows the predictive margins following a multinomial logistic regression conducted to determine the impact of the policy implementation on the disease stage at presentation. The predicted probabilities reveal significant variations in the probability of disease stage at presentation among gastric cancer patients, based on the interaction between the ACA implementation period and states' policy implementation status. Patients treated in New Jersey post-ACA implementation had the highest predicted probability of presenting with localized disease (36.1%, 95% CI: 34.1%-38.1%, p < 0.001) compared to those in Georgia in the post-ACA period (33.1%, 95% CI: 31.1%-35.2%, p < 0.001). Conversely, the probability of presenting with distant disease was relatively stable in New Jersey post-ACA (36.6%, 95% CI: 34.6%-38.6%, p < 0.001) compared to the pre-ACA period (36.7%, 95% CI: 35.4%-38.0%, p < 0.001). In Georgia, however, the predicted probability of presenting at the late stages of the disease slightly increased from 37.9% (95% CI: 36.4%-39.4%, p < 0.001) to 39.5% (95% CI: 37.4%-41.7%, p < 0.001) in the post-ACA period.

**Table 5:** Predicted probabilities of disease stages at presentation for patients with gastric cancer (SEER registry 2000-2020) The predicted probabilities were generated from a multinomial logistic regression after adjusting for patients' age, sex, race/ethnicity, marital status, and household median income ACA: Affordable Care Act; CI: Confidence interval; SEER: Surveillance, epidemiology, and end results

Variable	Margin	Standard error	z	p-value	Lower CI	Upper CI
Localized						
Georgia (pre-ACA)	0.306	0.007	42.12	<0.001	0.292	0.32
Georgia (post-ACA)	0.331	0.01	31.9	<0.001	0.311	0.352
New Jersey (pre-ACA)	0.3	0.006	46.92	<0.001	0.288	0.313
New Jersey (post-ACA)	0.361	0.01	35.3	<0.001	0.341	0.381
Regional						
Georgia (pre-ACA)	0.315	0.007	42.32	<0.001	0.301	0.33
Georgia (post-ACA)	0.273	0.01	27.46	<0.001	0.254	0.293
New Jersey (pre-ACA)	0.333	0.007	50.65	<0.001	0.32	0.346
New Jersey (post-ACA)	0.273	0.009	29.16	<0.001	0.255	0.292
Distant metastasis						
Georgia (pre-ACA)	0.379	0.008	49.22	<0.001	0.364	0.394
Georgia (post-ACA)	0.395	0.011	36.46	<0.001	0.374	0.417
New Jersey (pre-ACA)	0.367	0.007	54.52	<0.001	0.354	0.38
New Jersey (post-ACA)	0.366	0.01	35.71	<0.001	0.346	0.386

## Discussion

Our study found a substantive association between the implementation of the ACA and higher odds of early disease stage at presentation and higher survival (reduced mortality hazards) among individuals diagnosed with gastric cancers in New Jersey. While the mortality hazard decreased significantly post-ACA implementation in New Jersey and Georgia, the most substantial reduction was observed in New Jersey. Patients in New Jersey post-ACA were also more likely to present with localized disease compared to those in Georgia post-ACA. 

Our findings align with several studies that have documented improved cancer outcomes following the implementation of the ACA [15,17-19]. For instance, Jemal et al. [16] found that Medicaid expansion under the ACA was associated with increased early-stage diagnosis and reduced mortality in various cancers. Our results are consistent with these findings, particularly in the context of localized disease presentation and survival improvement in New Jersey.

However, other studies have reported mixed outcomes regarding the ACA's impact. A study by Salazar et al. [20] did not find significant differences in cancer survival rates between Medicaid expansion and non-expansion states. The discrepancies between our findings and those of Salazar et al. could be attributed to differences in study populations, cancer types examined, and methodological approaches [21].

Mechanisms and implications

The observed improvement in survival and early disease presentation in New Jersey post-ACA can be attributed to several mechanisms. The ACA facilitated increased insurance coverage, particularly through Medicaid expansion, which likely improved access to healthcare services [7,22]. This improved access may have led to earlier diagnosis and timely treatment, crucial factors in cancer prognosis. Additionally, New Jersey's healthcare infrastructure and policies may have better leveraged ACA provisions, enhancing patient outcomes.

The ACA's emphasis on preventive care and early detection programs [23-25] may also explain the higher rates of localized disease presentation in New Jersey post-ACA. Enhanced access to regular screenings and primary care services could lead to earlier detection of gastric cancer, which is typically more treatable in its early stages.

Future direction

While our study provides valuable insights into the positive impacts of the ACA, further research is needed to explore the long-term effects of the policy on various cancers across different states. Future studies should consider longitudinal designs to assess the sustained impact of the ACA on cancer outcomes. Additionally, research should investigate the specific components of the ACA that are most effective in improving cancer prognosis, which could inform future health policies.

Comparative studies between states with different levels of Medicaid expansion and healthcare infrastructure could also shed light on the contextual factors that enhance or hinder the ACA's effectiveness. Understanding these nuances will be crucial for policymakers aiming to optimize cancer care and outcomes across diverse populations.

Limitations and strengths

This study, which utilizes the SEER registry for a DiD analysis, has several limitations. First, the SEER database may not capture all relevant variables, such as detailed socioeconomic factors or individual health behaviors, which could confound the results. Second, variations in healthcare access and quality within states might not be fully accounted for, potentially affecting the generalizability of the findings. Lastly, the study period may not be long enough to capture the full impact of the ACA on long-term survival outcomes.

Despite these limitations, the study has notable strengths. The large, population-based SEER registry provides a robust sample size and comprehensive cancer data, enhancing the study's statistical power and external validity. The DiD design effectively controls for temporal trends, allowing for a clearer assessment of the ACA's impact. These strengths contribute to a meaningful evaluation of policy effects on cancer outcomes.

## Conclusions

Our study demonstrates that the ACA has had a significant positive impact on gastric cancer outcomes, particularly in states like New Jersey that fully embraced Medicaid expansion. The increased likelihood of early-stage disease presentation and improved survival rates highlight the importance of accessible healthcare services. As health policies continue to evolve, it is imperative to build on these findings to ensure equitable and effective cancer care for all patients. Future research should continue to evaluate the long-term benefits of the ACA and identify strategies to further enhance cancer care delivery. We recommend that policymakers prioritize the continuation and expansion of Medicaid services, as our findings suggest that such policies significantly enhance early cancer detection and improve survival rates, thereby underscoring the critical role of sustained governmental support in healthcare.
